# Polymorphisms in the gene encoding bovine interleukin-10 receptor alpha are associated with *Mycobacterium avium ssp. paratuberculosis *infection status

**DOI:** 10.1186/1471-2156-11-23

**Published:** 2010-04-15

**Authors:** Chris P Verschoor, Sameer D Pant, Qiumei You, Flavio S Schenkel, David F Kelton, Niel A Karrow

**Affiliations:** 1 Centre for Genetic Improvement of Livestock, Department of Animal and Poultry Science, University of Guelph, 50 Stone Rd East, Guelph, Ontario, N1G2W1, Canada; 2 Department of Population Medicine, University of Guelph, 50 Stone Rd. East, Guelph, Ontario, N1G2W1, Canada

## Abstract

**Background:**

Johne's disease is a chronic inflammatory bowel disease (IBD) of ruminants caused by *Mycobacterium avium *ssp. *paratuberculosis *(MAP). Since this pathogen has been implicated in the pathogenesis of human IBDs, the goal of this study was to assess whether single nucleotide polymorphism (SNPs) in several well-known candidate genes for human IBD are associated with susceptibility to MAP infection in dairy cattle.

**Methods:**

The bovine candidate genes, *interleukin-10 (IL10), IL10 receptor alpha/beta (IL10RA/B), transforming growth factor beta 1 (TGFB1)*, *TGFB receptor class I/II (TGFBR1/2)*, and *natural resistance-associated macrophage protein 1 (SLC11A1) *were sequenced for SNP discovery using pooled DNA samples, and the identified SNPs were genotyped in a case-control association study comprised of 242 MAP negative and 204 MAP positive Holstein dairy cattle. Logistic regression was used to determine the association of SNPs and reconstructed haplotypes with MAP infection status.

**Results:**

A total of 13 SNPs were identified. Four SNPs in *IL10RA *(984G > A, 1098C > T, 1269T > C, and 1302A > G) were tightly linked, and showed a strong additive and dominance relationship with MAP infection status. Haplotypes AGC and AAT, containing the SNPs *IL10RA *633C > A, 984G > A and 1185C > T, were associated with an elevated and reduced likelihood of positive diagnosis by serum ELISA, respectively.

**Conclusions:**

SNPs in *IL10RA *are associated with MAP infection status in dairy cattle. The functional significance of these SNPs warrants further investigation.

## Background

In ruminant livestock and some wild-life species, *Mycobacterium avium *ssp. *paratuberculosis *(MAP) causes Johne's disease, a chronic inflammatory bowel disorder (IBD) that parallels human Crohn's disease in many respects. Since MAP is a slow-growing intracellular pathogen, infected cattle typically remain asymptomatic for 2 to 10 years making it difficult to control Johne's disease in dairy herds [1]. During this asymptomatic period, the pathogen can be horizontally transmitted to other herd members via contaminated feces, and vertically transmitted to calves via contaminated milk and colostrum [1].

Although it is debatable, the presence of MAP in milk poses a potential zoonotic risk to humans [2]. This may be particularly relevant for individuals that are genetically predisposed to IBD, since MAP has been implicated as one of several potential pathogens associated with Crohn's disease [3]. A meta-analysis of studies examining the presence of MAP in patients with Crohn's disease or ulcerative colitis for example, showed that there was a greater likelihood of detecting MAP in diseased versus healthy individuals [4]. Additionally, clinical studies have also shown that anti-mycobacterial treatment of some patients with Crohn's disease can lead to pathological remission [5].

Variability in the susceptibility of cattle to MAP infection is evident. In a typical commercial dairy herd where there is a consistent prevalence of MAP infection for example, it is common to find animals that appear resistant to infection, even after several years of exposure. Additionally, there is evidence that susceptibility to MAP infection, and the development of clinical symptoms associated with Johne's disease is inherited; heritability estimates in dairy cattle have been estimated to range from 0.010 to 0.183, depending on the criteria used to diagnose MAP infection or Johne's disease [6-8]. Given this, it may be possible to use selective breeding strategies to enhance resistance to MAP infection thereby reducing the incidence of Johne's disease in dairy cattle and the risk of human exposure to MAP.

Since resistance to MAP infection is likely polygenic in nature, it is essential that multiple genes be investigated for their contribution to disease resistance. Therefore, the focus of this study was to identify single nucleotide polymorphisms (SNPs) in several immune-related genes and investigate their association with MAP infection status in dairy cattle. *Interleukin-10 (IL10) *and its receptor (subunits *IL10RA *and *IL10RB*), *transforming growth factor beta 1 (TGFB1) *and two of its receptors (*TGFBR1 and TGFBR2*), and *natural resistance-associated macrophage protein 1 *(*SLC11A1*) were investigated in this study based on their previous associations with various types of human IBD [9-12]. Interleukin-10 and TGFB1 collectively act to control the host inflammatory response to microbial antigens; IL10 primarily operates as a feedback inhibitor of T cell responses, and TGFB1's major function is to maintain T cell tolerance to self and commensal antigens by influencing the differentiation and homeostasis of effector and regulatory T cells [13]. Natural resistance-associated macrophage protein 1, also known as solute carrier family 11 member 1, is an iron transporter that exhibits pleiotropic effects on the early innate macrophage response to intracellular bacteria [14]. Of the 13 SNPs identified, four in *IL10RA *(984G > A, 1098C > T, 1269T > C, and 1302A > G) were tightly linked, and showed a strong additive and dominance relationship with MAP infection status.

## Methods

### Cohort population

Six commercial Holstein operations in Southwestern and Eastern Ontario were selected for sample collection based on a previous history of a high prevalence of MAP infection. Blood was collected between the months of July and September 2007 via the coccygeal (tail) vein from dry and lactating cows ranging in age, breed, stage of lactation, infection status, and history of MAP screening. The protocol for collection was approved by the University of Guelph animal care committee. Current infection status was determined by identifying the presence of MAP-specific plasma antibodies using the commercially available HerdChek M. pt. Antibody ELISA Test Kit (IDEXX Laboratories, Westbrook, ME, USA) according to manufacturer's instructions. A total of 380 cows were sampled, on average 63 ± 31 from each herd. Of which, at least 4% from each herd tested positive (S/P > 0.25) or suspect (0.10 < S/P < 0.25) of MAP-infection as diagnosed by serum ELISA. Suspect animals were not included in either cohort. Infection-free animals making up the healthy (negative) control cohort (n = 242) included animals that were older than 4.5 years of age and had tested negative for MAP infection in previous years (n = 197), and those that were older than 5.5 years of age without previous screening (n = 45). The mean age of this cohort was 6.4 years (range, 4.5 to 12.7 years). The infected (positive) cohort (n = 204) was made up of animals that were considered to be infected based on the presence of MAP-specific plasma antibodies (n = 16), and a second group of animals considered to be infected based on milk MAP-specific antibodies screening carried out by Canwest DHI (Guelph, ON, CAN) (n = 188); these milk samples were generously provided between July 2006 and November 2007, and due to client anonymity, information such as age, pedigree and herd location was not available. Genomic DNA was extracted from the buffy coat of blood samples using the DNeasy blood and tissue kit (Qiagen, Santa Clara, CA, USA), and from milk according to methods previously described [15].

### SNP discovery

All SNPs were identified by sequencing PCR amplicons from each candidate gene using a DNA pool constructed with DNA from 40 Holstein bulls according to methods described in previous studies [16,17]. Briefly, for each bull, genomic DNA was extracted from semen and adjusted to a concentration of 5 ng/μl after several rounds of quantification using the Quant-iT PicoGreen dsDNA reagent (Invitrogen, Carlsbad, CA, USA) followed by dilution. The resultant DNA pool was amplified using the Repli-g Ultrafast mini kit (Qiagen, Santa Clara, CA, USA), and was then used as a template for PCR amplification of the 5' region and coding exons of each candidate gene. Primers were designed using Primer3 [18]. PCR amplicons were sequenced in both 5' and 3' orientation using an ABI Prism 3730 DNA sequencer (Applied Biosystems, Foster City CA, USA), and SNPs were identified by visual inspection of the electropherograms. Seven genes were selected for SNP discovery, *IL10 *[GeneId#, 281246], *IL10RA *[513478] and *IL10RB *[767864], *TGFB*1 [282089], *TGFBR1 *[282382] and *TGFBR2 *[535376], and *SLC11A1 *[282470]. Sequences were compared against GLEAN models using the Apollo Genome Annotation and Curation Tool to confirm correct gene structure (Version 1.6.5) [19]. In the event of a disagreement between respective GLEAN and NCBI gene models, as was the case for *IL10RA*, the GLEAN model was chosen.

### Genotyping and haplotype reconstruction

SNP genotyping was conducted using the iPLEX MassARRAY system (Sequenom inc., San Diego, CA, USA). Two of the thirteen SNPs, *IL10 *-30A > C, and *SLC11A1 *650C > T, were not genotyped using this assay due to failed primer design or inadequate quality of results (Table 1). Two groups of SNPs, *IL10RA *984G > A, 1098C > T, 1269T > C and 1302A > G, and *IL10RB *503C > T and 569A > G appeared to be tightly linked due to nearly matching genotype records (Pearson's r ≥ 99%), thus all but one SNP from each group was removed from the analysis. For haplotype analysis, only the SNPs in *IL10RA *were included since no other genes contained multiple informative SNPs, or resided on the same chromosome. The haplotypes were reconstructed in both cohorts using PHASE (version 2.1) [20].

**Table 1:** Characteristics of SNPs discovered in *IL10*, *IL10RA*/*B*, *TGFB1*, and *SLC11A1*.

Gene	SNP	dbSNP ssID	Region	Mutation	Primer set (5'-3')
*IL10*	-30A > C	ss104807640	5'		F: GGTAAAGCAGTCCTGAATCCAA
					R: TCCTTCATGGGCCCTATTT
	-285T > C	ss104807641	5'		F: AGCCAGCAGCTCTCAAAGTC
					R: GTGTTCAGTGTGGTCCTGGAT
	
*IL10RA*	633C > A	ss104807642	Coding	Syn	F: TCGTGTTTATTGCTCTGGTTGT
					R: CCTGCTTCCTTCCCTCCT
	984G > A^a^	ss104807643	Coding	Syn	F: GGGTTCCTGCTGGTGACTC
					R: GCCAATGCCACTGTCCTC
	1098C > T^a^	ss104807644	Coding	Syn	F: GGGTTCCTGCTGGTGACTC
					R: GCCAATGCCACTGTCCTC
	1185C > T	ss104807645	Coding	Syn	F: AGTGCAGACAGCGGGATCT
					R: TTCTTCAGGGGTCTGCAAAG
	1269T > C^a^	ss104807646	Coding	Syn	F: AGTGCAGACAGCGGGATCT
					R: TTCTTCAGGGGTCTGCAAAG
	1302A > G^a^	ss104807647	Coding	Syn	F: AGTGCAGACAGCGGGATCT
					R: TTCTTCAGGGGTCTGCAAAG
	
*IL10RB*	503C > T^b^	ss104807648	Coding	Non	F: GGGAATTCAGGGAATAAAGCA
					R: CTGTTTGGGGAATGCAGATT
	569A > G^b^	ss104807649	Coding	Non	F: GGGAATTCAGGGAATAAAGCA
					R: CTGTTTGGGGAATGCAGATT
	
*TGFB1*	258C > T	ss104807650	Coding	Syn	F: CCCTTGCCAAACACTGACA
					R: CCTAGCCCAGGCCACTTT
	
*SLC11A1*	650C > T	ss104807654	Coding	Non	F: TCCTCTGGAGAAGGGAAAGG
					R: ATTCAGAGGCAGGAGTCGAG
	1066C > G	ss104807655	Coding	Non	F: ACATGTGTTGGCCAAGTGAA
					R: ACATCCGAGTCCTGAGTGGT

Syn/Non, synonymous/non-synonymous; F/R, forward/reverse primers.

^a, b ^SNPs with common superscripts are tightly linked (Pearson's r ≥ 99%).

### Statistical analysis

Tests for significance of pair-wise linkage disequilibrium were performed as described in Krawetz and Womble [21]:

where: *η *= number of cows genotyped; *ρ*_AB _= frequency of haplotype AB; *ρ*_A_, *ρ*_a _= frequency of alleles A and a, respectively; *ρ*_B_, *ρ*_b _= frequency of alleles B and b, respectively.

SNP associations were determined using a logistic regression approach (PROC LOGISTIC) in SAS (version 9.1, SAS Institute Inc., NC, USA) using the following model:

where: *y*_*ij *_= MAP infection status (1 = infected, 0 = healthy) for the *j-*th cow; *μ *= overall mean; *s *= number of SNPs on the particular chromosome considered; *a*_*i *_= additive effect for the *i-*th SNP; *w*_*i *_= genotype of the *i-*th SNP recoded as number of alleles (0, 1 and 2); *d*_*i*_= dominance effect for the *i-*th SNP; *v*_*i *_= genotype of the *i-*th SNP recoded as homozygote or heterozygote (0 and 1); *a*_*k *_= additive effect for the k-th null SNP; *m*_*k *_= genotype of k-th null SNP recoded as number of alleles (0, 1 and 2); and *e*_*ij *_= random residual effect for the *j*-th cow. Four null SNPs were included as covariates in each logistic regression model as a means to correct for bias that may arise due to stratification between the negative and positive cohorts [22]. These SNPs are unlinked with respect to one another and to all candidate SNPs considered, are not expected to be associated with any traits related to MAP-infection, and are polymorphic within the Canadian Holstein population (minor allele frequency (MAF) > 35%) (unpublished results).

Haplotype analysis was performed in SAS using a similar model, only  is replaced by ; where: *β*_*i *_= linear regression coefficient (haplotype effect) for the *i*-th haplotype; *Hap*_*ij *_= the probability for the *i*-th haplotype for the *j*-th cow, where *h *is the number of observed haplotypes.

Experimental-wise significance levels for all tests were determined by Bonferroni correction. Regression results are presented as odds ratios (OR) and their respective 95% confidence intervals (CI).

To assess the level of multicollinearity among the SNPs included in the analysis, principal component analysis (PCA) was performed using PROC PRINCOMP in SAS, followed by calculation of the condition index [23]:

where: *λ*_max_, *λ*_min _= the largest and smallest eigenvalue for the variables considered, respectively. Akaike's information criterion (AIC) was used to select the final regression model when multicollinearity was shown to be unacceptable.

## Results

In total, thirteen SNPs were identified: two in *IL10 *[-285T > C (dbSNP ssID# ss104807640) and -30A > C (ss104807641)]; six in *IL10RA *[633C > A (ss104807642), 984G > A (ss104807643), 1098C > T (ss104807644), 1185C > T (ss104807645), 1269T > C (ss104807646), and 1302A > G (ss104807647)]; two in *IL10RB *[503C > T (ss104807648), and 569A > G (ss104807649)], one in *TGFB1 *[258C > T (ss104807650)], and two in *SLC11A1 *[650C > T (ss104807654) and 1066C > G (ss104807655)]. All SNPs were submitted to NCBI dbSNP (Build 130).

Due to nearly matching genotype distributions (Pearson's r ≥ 99%) it was assumed that four SNPs in *IL10RA *(984G > A, 1098C > T, 1269T > C and 1302A > G), and both SNPs in *IL10RB *(503C > T and 569A > G) were tightly linked. Hence, all but one SNP from each of these genes was dropped from analysis in order to minimize redundancy. Pair-wise linkage disequilibrium (r^2^) estimates for the remaining SNPs in *IL10RA *were 0.07 and 0.41, between SNPs *IL10RA *633C > A and 1185C > T, and 984G > A and 1185C > T, respectively, and significant at p < 0.001; similar results were previously reported in Canadian Holstein bulls [24]. As such, it was a concern that there would be a high degree of correlation (multi-collinearity) between them in the present dataset, thereby inflating standard error of parameter estimates and thus, reducing the significance of resultant associations [25]. Principal component analysis (PCA), followed by calculation of the condition index, suggested that these three SNPs were in a state of strong multi-collinearity (K > 140), whereas the removal of any one SNP returned the condition index to an acceptable range (7.7 < K < 10.5) [26]. Model selection based on AIC subsequently determined that *IL10RA *1185C > T was the most appropriate SNP to remove from the final multiple regression model.

Logistic regression analysis revealed that only the SNPs in *IL10RA *were associated with MAP infection. The group of tightly linked SNPs, *IL10RA *984G > A, 1098C > T, 1269T > C and 1302A > G, were found to have a strong additive and dominance relationship with MAP infection status (OR, 0.51 (0.34-0.78), p = 0.002, and 2.27 (1.40-3.67), p = 0.001, respectively), which were retained at an experimental-wise significance of 5% (Table 2). The results suggest that the allele GCTA is dominant over the ATCG allele, and is associated with a higher probability of being positive for MAP. The SNP *IL10RA *633C > A also showed modest additive relationship with MAP infection status (OR, 0.54 (0.29-1.00), p = 0.049), in which the C allele is associated with a higher probability of being positive for MAP.

**Table 2:** Genotypic frequencies and associations of SNPs in *IL10*, *IL10RA*/*B*, *TGFB1*, and *SLC11A1 *with MAP-infection status.

			MAP infection status			
			Negative	Positive		
					
Gene	SNP	Genotype	# (%)	# (%)	Effect ± SE	OR (CI)
*IL10*	-285T > C	n	208	178	*α*: -0.29 ± 0.28	0.75 (0.43-1.29)
		TT	163 (78.4)	136 (76.4)	*δ*: 0.45 ± 0.37	1.58 (0.76-3.29)
		CT	34 (16.3)	37 (20.8)		
		CC	11 (5.3)	5 (2.8)		
	
*IL10RA*	633C > A	n	238	193	*α*: -0.62 ± 0.32*	0.54 (0.29-1.00)
		CC	10 (4.2)	7 (3.6)	*δ*: -0.54 ± 0.36	0.58 (0.29-1.18)
		CA	72 (30.3)	54 (28)		
		AA	156 (65.5)	132 (68.4)		
	984G > A	n	235	183	*α*: -0.67 ± 0.22**	0.51 (0.34-0.78)
		AA	56 (23.8)	18 (9.8)	*δ*: 0.82 ± 0.25**	2.27 (1.40-3.67)
		AG	111 (47.2)	109 (59.6)		
		GG	68 (28.9)	56 (30.6)		
	1185C > T	n	240	198	*rm*	*rm*
		TT	18 (7.5)	5 (2.5)		
		TC	93 (38.8)	75 (37.9)		
		CC	129 (53.8)	118 (59.6)		
	
*IL10RB*	503C > T	n	216	182	*α*: 0.27 ± 0.17	1.31 (0.95-1.81)
		CC	36 (16.7)	22 (12.1)	*δ*: 0.07 ± 0.22	1.08 (0.69-1.67)
		TC	103 (47.7)	89 (48.9)		
		TT	77 (35.6)	71 (39)		
	
*TGFB1*	258C > T	n	237	201	*α*: 0.08 ± 0.16	1.08 (0.80-1.47)
		CC	98 (41.4)	74 (36.8)	*δ*: 0.04 ± 0.22	1.04 (0.68-1.58)
		CT	106 (44.7)	97 (48.3)		
		TT	33 (13.9)	30 (14.9)		
	
*SLC11A1*	1066C > G	n	219	176	*α*: -0.27 ± 0.37	0.77 (0.37-1.58)
		CC	142 (64.8)	106 (60.2)	*δ*: 0.45 ± 0.41	1.57 (0.70-3.50)
		CG	71 (32.4)	67 (38.1)		
		GG	6 (2.7)	3 (1.7)		

# (%), genotypic count (frequency); Effect ± SE, additive (*α*) or dominance (*δ*) effect ± standard error; OR (95% CI), odds ratio (95% confidence interval); rm, removed due to strong multi-collinearity.

* Comparison-wise significance at 5% from logistic regression.

** Experimental-wise significance at 5% from logistic regression and Bonferroni's procedure for multiple testing correction.

Haplotype reconstruction of the three informative SNPs in *IL10RA *(633C > A, 984G > A and 1185C > T) identified four combinations: AGC, AAT, CAC and AAC. Haplotype AAC was found in less than 1% of the sample population, whereas haplotypes AGC, AAT and CAC represented 56%, 24% and 19% of the entire sample population, respectively (Table 3). Individual tests for haplotype association with MAP infection revealed that haplotype AGC was associated with a higher probability of being positive for MAP (p = 0.018) and haplotype AAT with a higher probability of being negative for MAP (p = 0.030) (Table 3). Haplotype contrasts against the most frequent haplotype, AGC, identified a significant effect for haplotype AAT (p = 0.013)(Table 3), which was retained at an experimental-wise level of 5%.

**Table 3:** Haplotype frequencies in the 3' coding region of *IL10RA *and their association with MAP-infection status.

*IL10RA *haplotype			Frequency				
			
			MAP infection status				
633C > A	984G > A	1185C > T	Negative (n = 235)	Positive (n = 180)	*β *± SE	OR (95% CI)	Contrast ± SE
A	G	C	52.6%	60.6%	0.35 ± 0.15 *	1.42 (1.06-1.90)	
A	A	T	27.2%	20.8%	-0.37 ± 0.17 *	0.69 (0.49-0.97)	-0.45 ± 0.18 **
C	A	C	19.1%	17.8%	-0.09 ± 0.18	0.92 (0.65-1.30)	-0.23 ± 0.19
A	A	C	1.1%	0.8%	-0.25 ± 0.74	0.78 (0.18-3.31)	-0.47 ± 0.74

*β *± SE, haplotype effect ± standard error; OR (95% CI), odds ratio (95% confidence interval); contrast ± SE, haplotype contrast ± standard error against the most frequent haplotype, AGC.

* Comparison-wise significance at 5% probability level from logistic regression of haplotype counts against infection status.

* * Experimental-wise significance at 5% probability level from logistic regression and Bonferroni's correction for multiple hypothesis testing.

## Discussion

In the following cohort study, two significant associations with MAP infection status were observed for the *IL10RA *gene. First, a strong association between the tightly linked bovine SNPs *IL10RA *984G > A, 1098C > T, 1269T > C and 1302A > G and MAP infection status was detected. For these SNPs, the allele GCTA is dominant over the ATCG allele, and is associated with a higher probability of being positive for MAP. Second, when haplotype analysis was performed on SNPs *IL10RA *633C > A, 984G > A and 1185C > T, equally strong, inverse associations for the haplotypes AGC and AAT with MAP infection status were observed. Considering the strong individual relationship of *IL10RA *984G, 1098C, 1269T and 1302A, with MAP infection status, it is not unreasonable to assume that these SNPs are the primary contributor to these associations. Furthermore, removing 633C > A from the haplotype reconstruction resulted in haplotype frequencies and associations that were negligibly different from those reported above (unpublished results). Contrasts indicated a strong, significant effect in reducing the proportion of infected animals when replacing the most frequent haplotype, AGC, with AAT. This would suggest that it may be possible to lower the overall frequency of MAP infection as diagnosed by serum ELISA by increasing the frequency of the AAT haplotype through selective breeding. However, since the impact of such a breeding decision on the prevalence of other diseases is currently unknown, such a strategy should be approached with caution.

Interleukin-10 has emerged as an essential immunoregulatory cytokine during bacterial infections. In the context of *Mycobacterium *spp. for example, IL10 helps to control excessive T helper 1 and CD8^+ ^T cell responses that contribute to the immunopathology associated with infection; it also prevents the overproduction of IL4, IL5, and IL13, which can lead to severe fibrosis during the T helper 2 response [27]. This may be particularly relevant at mucosal surfaces, since human studies have implicated functional SNPs in the *IL10 *gene as risk factors for IBD [10] and tuberculosis [28]. In cattle, *IL10 *is up-regulated during subclinical and clinical MAP infections [29,30], and its neutralization has been shown to promote the activation of MAP-infected bovine macrophages and subsequent killing of the organism [31]. Similar findings have also been demonstrated with human infection studies performed *in vitro *using *Mycobacterium tuberculosis *[32,33].

Although the present study found no association between variants in the bovine *IL10 *gene and MAP infection, it did provide evidence that variants in the *IL10RA *gene, which encodes the ligand-binding subunit of the IL10R and is a major determinant of IL10 responsiveness [34,35], may contribute to susceptibility to MAP infection. We are unaware of previous studies indicating that variants in the *IL10R *gene influence the susceptibility to mycobacterium infection. However, previous work has shown that antibody-mediated neutralization of the IL10R complex reduces the susceptibility of mice to *Mycobacterium avium *and increase the efficacy of anti-mycobacterial therapy [36]. In support of this, associations have been reported between SNPs in the human *IL10RA *and *IL10RB *genes and the level of *IL10 *expression in mucosal tissues [37]. Our group has also recently identified associations in Holstein dairy bulls between the presently studied *IL10RA *haplotype and estimated breeding values for somatic cell score, a trait highly correlated to the incidence of the inflammatory disease mastitis [24]. Furthermore, based on alignment with the murine homologue, all of the SNPs identified within *IL10RA*, with exception to 633C > A, appear to code for a region of the cytoplasmic domain that defines cellular responsiveness to IL10 and mediates cellular proliferation [38]. It is important to note, however, that before any definite conclusions can be made concerning the magnitude of *IL10RA*'s contribution to susceptibility/resistance to MAP infection, it will be necessary to functionally characterize the *IL10RA *haplotype, since the SNPs it represents are all synonymous mutations that may be linked to a nearby gene on BTA15 that confers susceptibility/resistance to MAP infection. Traditionally, synonymous SNPs are viewed as "silent" and thus may not warrant functional validation, however, several studies addressing the role of codon usage bias, as well as mRNA folding, have reported otherwise [39-41].

Two limitations related to the design of the present study warrant that the reported associations be approached with caution. First, the cows in the negative cohort were categorized as such using the serum ELISA, and in some cases, the milk ELISA as well. These tests, while known to be very specific, have relatively low sensitivities; 25-94% and 29-61%, for the serum and milk ELISA, respectively [42]. Therefore, it is likely a proportion of negative animals are in fact MAP infected, but have yet to produce a strong enough antibody-response required for positive diagnosis. A major implication is that such bias could skew allelic frequencies for certain SNPs, leading to an under- or over-estimation of the true effect. In the present study, the control cohort consisted of cows older than 4.5 years of age (mean = 6.4 years) that had been diagnosed negative by serum ELISA, the majority of which with previous screening by milk ELISA. Given that it is generally accepted that cattle become MAP-infected as calves [43], we believe that a cohort consisting of older cows will likely contain a sufficiently low number of false negatives, thereby minimizing any related bias. This is supported by a number of studies reporting a positive correlation between seroprevalence and age [44-46].

The second limitation of the present study was the absence of information about the family structure of a substantial number of the genotyped cows; this prevented us from classifying population stratification and substructure, which can bias genetic association studies [47]. To address this issue, we included null markers as covariates in the logistic regression SNP association analysis. This approach has be shown to be very effective for controlling bias due to stratification in candidate gene association studies [22], and whole genome association approaches [48]. While these SNPs are suitable for inclusion as null markers since they are unlinked to one another and to the candidate SNPs considered, and are polymorphic within the Canadian Holstein population (MAF > 35%), they have not been validated for their ability to identify stratification within said population. Thus, it is essential that the results presented in this study are validated in a larger and more diverse cohort study in order to ensure that the associations drawn from the present study are not skewed by biasing effects such as family structure.

## Conclusions

In summary, several SNPs were identified in the bovine genes encoding *IL10*, *IL10RA*, *IL10RB*, *TGFB1*, and *SLC11A1*. A strong association between a group of tightly linked synonymous SNPs in the 3' coding region of *IL10RA*, 984G > A, 1098C > T, 1269T > C and 1302A > G, and MAP infection status Canadian dairy cattle was established. Haplotype reconstruction of the SNPs in *IL10RA *also revealed a strong association with MAP infection status. These results provide initial evidence that variants in *IL10RA *may contribute to susceptibility to MAP infection in dairy cattle.

## Competing interests

The authors declare that they have no competing interests.

## Authors' contributions

CPV designed the study, collected blood samples, extracted DNA, performed SNP discovery and all analyses, and prepared the manuscript. SDP collected milk samples, extracted DNA, and aided in the statistical design. QY performed the MAP serum ELISAs. FSS supervised all statistical analyses and revised the manuscript. DFK aided in obtaining the HerdChek M. pt. Antibody ELISA Test Kit, and revised the manuscript. NAK provided funding and supervised all aspects of the study, and approved the final manuscript. All authors read and approved the final manuscript.

## References

[B1] McKenna SL Keefe GP Tiwari A VanLeeuwen J Barkema HW Johne's disease in Canada part II: disease impacts, risk factors, and control programs for dairy producers Can Vet J 2006 47 1089 1099 17147140 PMC1624920

[B2] Waddell LA Rajic A Sargeant J Harris J Amezcua R Downey L The zoonotic potential of Mycobacterium avium spp. paratuberculosis: a systematic review Can J Public Health 2008 99 145 155 18457292 10.1007/BF03405464 PMC6975835

[B3] Glasser AL Darfeuille-Michaud A Abnormalities in the handling of intracellular bacteria in Crohn's disease: a link between infectious etiology and host genetic susceptibility Arch Immunol Ther Exp (Warsz) 2008 56 237 244 10.1007/s00005-008-0026-1 18726145

[B4] Feller M Huwiler K Stephan R Altpeter E Shang A Furrer H Mycobacterium avium subspecies paratuberculosis and Crohn's disease: a systematic review and meta-analysis Lancet Infect Dis 2007 7 607 613 10.1016/S1473-3099(07)70211-6 17714674

[B5] Chamberlin W Ghobrial G Chehtane M Naser SA Successful treatment of a Crohn's disease patient infected with bacteremic Mycobacterium paratuberculosis Am J Gastroenterol 2007 102 689 691 10.1111/j.1572-0241.2007.01040_7.x 17335456

[B6] Koets AP Adugna G Janss LL van Weering HJ Kalis CH Wentink GH Genetic variation of susceptibility to Mycobacterium avium subsp. paratuberculosis infection in dairy cattle J Dairy Sci 2000 83 2702 2708 10.3168/jds.S0022-0302(00)75164-2 11104291

[B7] Gonda MG Chang YM Shook GE Collins MT Kirkpatrick BW Genetic variation of Mycobacterium avium ssp. paratuberculosis infection in US Holsteins J Dairy Sci 2006 89 1804 1812 10.3168/jds.S0022-0302(06)72249-4 16606752

[B8] Mortensen H Nielsen SS Berg P Genetic variation and heritability of the antibody response to Mycobacterium avium subspecies paratuberculosis in Danish Holstein cows J Dairy Sci 2004 87 2108 2113 10.3168/jds.S0022-0302(04)70029-6 15328223

[B9] Tamizifar B Lankarani KB Naeimi S Rismankar ZM Taghavi A Ghaderi A Promoter polymorphism of transforming growth factor-beta1 gene and ulcerative colitis World J Gastroenterol 2008 14 243 247 10.3748/wjg.14.243 18186562 PMC2675121

[B10] Tedde A Laura PA Bagnoli S Congregati C Milla M Sorbi S Interleukin-10 promoter polymorphisms influence susceptibility to ulcerative colitis in a gender-specific manner Scand J Gastroenterol 2008 43 712 718 10.1080/00365520701885507 18569989

[B11] Sechi LA Rosu V Pacifico A Fadda G Ahmed N Zanetti S Humoral immune responses of type 1 diabetes patients to Mycobacterium avium subsp. paratuberculosis lend support to the infectious trigger hypothesis Clin Vaccine Immunol 2008 15 320 326 10.1128/CVI.00381-07 18077612 PMC2238046

[B12] Zaahl MG Winter TA Warnich L Kotze MJ The -237C-->T promoter polymorphism of the SLC11A1 gene is associated with a protective effect in relation to inflammatory bowel disease in the South African population Int J Colorectal Dis 2006 21 402 408 10.1007/s00384-005-0019-z 16059695

[B13] Li MO Flavell RA Contextual regulation of inflammation: a duet by transforming growth factor-beta and interleukin-10 Immunity 2008 28 468 476 10.1016/j.immuni.2008.03.003 18400189

[B14] McDermid JM Prentice AM Iron and infection: effects of host iron status and the iron-regulatory genes haptoglobin and NRAMP1 (SLC11A1) on host-pathogen interactions in tuberculosis and HIV Clin Sci (Lond) 2006 110 503 524 10.1042/CS20050273 16597321

[B15] Murphy MA Shariflou MR Moran C High quality genomic DNA extraction from large milk samples J Dairy Res 2002 69 645 649 10.1017/S0022029902005848 12463700

[B16] Pant SD Schenkel FS Leyva-Baca I Sharma BS Karrow NA Identification of single nucleotide polymorphisms in bovine CARD15 and their associations with health and production traits in Canadian Holsteins BMC Genomics 2007 8 421 10.1186/1471-2164-8-421 18005441 PMC2234259

[B17] Sharma BS Leyva I Schenkel F Karrow NA Association of toll-like receptor 4 polymorphisms with somatic cell score and lactation persistency in Holstein bulls J Dairy Sci 2006 89 3626 3635 10.3168/jds.S0022-0302(06)72402-X 16899698

[B18] Rozen S Skaletsky H Primer3 on the WWW for general users and for biologist programmers Methods Mol Biol 2000 132 365 386 10547847 10.1385/1-59259-192-2:365

[B19] Lewis SE Searle SM Harris N Gibson M Lyer V Richter J Apollo: a sequence annotation editor Genome Biol 2002 3 RESEARCH0082 10.1186/gb-2002-3-12-research0082 12537571 PMC151184

[B20] Stephens M Donnelly P A comparison of bayesian methods for haplotype reconstruction from population genotype data Am J Hum Genet 2003 73 1162 1169 10.1086/379378 14574645 PMC1180495

[B21] Krawetz SA Womble DD Introduction to Bioinformatics: A Theoretical and Practical Approach 2003 Totowa, NJ, Humana Press

[B22] Wang Y Localio R Rebbeck TR Bias correction with a single null marker for population stratification in candidate gene association studies Hum Hered 2005 59 165 175 10.1159/000085940 15920341

[B23] Belsley DA Conditioning Diagnostics: Collinearity and Weak Data in Regression 1991 1 NY, USA: Wiley-Interscience

[B24] Verschoor CP Pant SD Schenkel FS Sharma BS Karrow NA SNPs in the bovine IL10 receptor are associated with somatic cell score in Canadian dairy bulls Mamm Genome 2009 20 447 454 10.1007/s00335-009-9198-1 19641966

[B25] Slinker BK Glantz SA Multiple regression for physiological data analysis: the problem of multicollinearity Am J Physiol 1985 249 4014489 10.1152/ajpregu.1985.249.1.R1

[B26] Meloun M Militky J Hill M Brereton RG Crucial problems in regression modelling and their solutions Analyst 2002 127 433 450 10.1039/b110779h 12022637

[B27] Couper KN Blount DG Riley EM IL10: the master regulator of immunity to infection J Immunol 2008 180 5771 5777 18424693 10.4049/jimmunol.180.9.5771

[B28] Ates O Musellim B Ongen G Topal-Sarikaya A Interleukin-10 and tumor necrosis factor-alpha gene polymorphisms in tuberculosis J Clin Immunol 2008 28 232 236 10.1007/s10875-007-9155-2 18071881

[B29] Karcher EL Beitz DC Stabel JR Modulation of cytokine gene expression and secretion during the periparturient period in dairy cows naturally infected with Mycobacterium avium subsp. paratuberculosis Vet Immunol Immunopathol 2008 123 277 288 10.1016/j.vetimm.2008.02.006 18374424

[B30] Khalifeh MS Stabel JR Upregulation of transforming growth factor-beta and interleukin-10 in cows with clinical Johne's disease Vet Immunol Immunopathol 2004 99 39 46 10.1016/j.vetimm.2004.01.009 15113652

[B31] Weiss DJ Evanson OA de SC Abrahamsen MS A critical role of interleukin-10 in the response of bovine macrophages to infection by Mycobacterium avium subsp paratuberculosis Am J Vet Res 2005 66 721 726 10.2460/ajvr.2005.66.721 15900955

[B32] Fietta A Meloni F Francioli C Morosini M Bulgheroni A Casali L Virulence of Mycobacterium tuberculosis affects interleukin-8, monocyte chemoattractant protein-1 and interleukin-10 production by human mononuclear phagocytes Int J Tissue React 2001 23 113 125 11771775

[B33] Al-Attiyah R Mustafa AS Characterization of human cellular immune responses to novel Mycobacterium tuberculosis antigens encoded by genomic regions absent in Mycobacterium bovis BCG Infect Immun 2008 76 4190 4198 10.1128/IAI.00199-08 18573897 PMC2519442

[B34] Ding Y Qin L Zamarin D Kotenko SV Pestka S Moore KW Differential IL10R1 expression plays a critical role in IL10-mediated immune regulation J Immunol 2001 167 6884 6892 11739506 10.4049/jimmunol.167.12.6884

[B35] Tamassia N Calzetti F Menestrina N Rossato M Bazzoni F Gottin L Circulating neutrophils of septic patients constitutively express IL10R1 and are promptly responsive to IL10 Int Immunol 2008 20 535 541 10.1093/intimm/dxn015 18308712

[B36] Roque S Nobrega C Appelberg R Correia-Neves M IL10 underlies distinct susceptibility of BALB/c and C57BL/6 mice to Mycobacterium avium infection and influences efficacy of antibiotic therapy J Immunol 2007 178 8028 8035 17548640 10.4049/jimmunol.178.12.8028

[B37] Simhan HN Ryckman KK Williams SM Krohn MA Genetic regulation of cervical antiinflammatory cytokine concentrations during pregnancy Am J Obstet Gynecol 2008 199 163 18674658 10.1016/j.ajog.2008.02.033

[B38] Ho AS Wei SH Mui AL Miyajima A Moore KW Functional regions of the mouse interleukin-10 receptor cytoplasmic domain Mol Cell Biol 1995 15 5043 5053 7544437 10.1128/mcb.15.9.5043 PMC230751

[B39] Duan J Wainwright MS Comeron JM Saitou N Sanders AR Gelernter J Synonymous mutations in the human dopamine receptor D2 (DRD2) affect mRNA stability and synthesis of the receptor Hum Mol Genet 2003 12 205 216 10.1093/hmg/ddg055 12554675

[B40] Chamary JV Hurst LD Evidence for selection on synonymous mutations affecting stability of mRNA secondary structure in mammals Genome Biol 2005 6 R75 10.1186/gb-2005-6-9-r75 16168082 PMC1242210

[B41] Salomons GS Bok LA Struys EA Pope LL Darmin PS Mills PB An intriguing "silent" mutation and a founder effect in antiquitin (ALDH7A1) Ann Neurol 2007 62 414 418 10.1002/ana.21206 17721876

[B42] Nielsen SS Toft N Ante mortem diagnosis of paratuberculosis: a review of accuracies of ELISA, interferon-gamma assay and faecal culture techniques Vet Microbiol 2008 129 217 235 10.1016/j.vetmic.2007.12.011 18255239

[B43] Windsor PA Whittington RJ Evidence for age susceptibility of cattle to Johne's disease Vet J 2009 184 1 37 44 10.1016/j.tvjl.2009.01.007 19246220

[B44] Geisbauer E Khol JL Wassertheurer M Damoser J Osterreicher E Dunser M Longterm investigation in an Austrian dairy herd with low prevalence of paratuberculosis detection of antibodies in blood and milk Vet Q 2007 29 138 148 18265703 10.1080/01652176.2007.9695239

[B45] Nielsen SS Toft N Age-specific characteristics of ELISA and fecal culture for purpose-specific testing for paratuberculosis J Dairy Sci 2006 89 569 579 10.3168/jds.S0022-0302(06)72120-8 16428626

[B46] Woodbine KA Schukken YH Green LE Ramirez-Villaescusa A Mason S Moore SJ Seroprevalence and epidemiological characteristics of Mycobacterium avium subsp. paratuberculosis on 114 cattle farms in south west England Prev Vet Med 2009 89 102 109 10.1016/j.prevetmed.2009.02.005 19327856

[B47] Rodriguez-Murillo L Greenberg DA Genetic association analysis: a primer on how it works, its strengths and its weaknesses Int J Androl 2008 31 546 556 10.1111/j.1365-2605.2008.00896.x 18522673

[B48] Setakis E Stirnadel H Balding DJ Logistic regression protects against population structure in genetic association studies Genome Res 2006 16 290 296 10.1101/gr.4346306 16354752 PMC1361725

